# Supplementary material for: Thermal Stability and Matrix Binding of Citrinin in the Thermal Processing of Starch-Rich Foods
Source: Toxins (Basel). 2025 Feb 13;17(2):86. doi: 10.3390/toxins17020086 (PMC11860567; doi:10.3390/toxins17020086)
Supplement: Supplementary file 1 [file toxins-17-00086-s001.zip › toxins-3407646-supplementary.pdf]

Supplementary Materials for

Thermal stability and matrix binding of citrinin in the thermal processing of starch-rich foods

Lea Brückner, Florian Neuendorff, Katharina Hadenfeldt, Matthias Behrens, Benedikt Cramer* and Hans-Ulrich Humpf*

Institute of Food Chemistry, University of Münster, 48149 Münster, Germany

*Corresponding author: humpf@uni-muenster.de; cramerb@uni-muenster.de

Optimization of the starch digestion - Sample preparation and analysis using GC-MS

To optimize starch digestion, 1 g of wheat flour (type 405) was weighed into a 10 mL screw-cap centrifuge glass tube, and digestion parameters were varied as described below. The sample was suspended in a defined volume (2 mL/ 4 mL) of water or sodium acetate buffer 50 mM. Heat-stable α-amylase from *Bacillus sp*. (100 µL/ 200 µL) was added to the suspension, and the samples were shaken intensively for 30 sec. The samples were incubated for different times (0.5, 1, 12 h) and temperatures (40, 65, 95 °C). Subsequently, 100 µL of an amyloglucosidase solution (from *Aspergillus niger)* (0.1, 1, 10 mg/mL) was added. Two suppliers (Sigma-Aldrich, Steinheim, Germany; Merck KGaAm, Darmstadt, Germany) were compared. The samples were incubated at 55 °C for different times (1, 2, 19, 24 h) while gently shaking (200 rpm, GFL type 3005, Gesellschaft für Labortechnik mbH, Burgwedel, Germany).

The result of each digestion was checked using GC-MS after oximation and silylation. The total ion chromatogram (TIC) was used to assess the quality of the digestion. Digestion could be considered quantitative if only an α-D-glucose peak, but no maltose peak or other sugars, was detected. For oximation and silylation, the samples were treated as follows. The samples were centrifuged (2370 *g*, 5 min, rt) (Universal 320 R, Andreas Hettich GmbH, Tuttlingen, Germany), and 200 µL of the supernatant was diluted in 70 % methanol to a volume of 10 mL. After repeated centrifugation (2370 *g*, 5 min, rt) (Universal 320 R, Andreas Hettich GmbH), 100 µL of the supernatant was pipetted into a 1.5 mL vial and dried completely at 40 °C under a stream of compressed air. To the residue, 100 µL of oximation reagent (1 % hydroxylammonium chloride (99 %, ThermoFisher GmbH, Kandel, Germany) in dry pyridine (≥99 %, Carl Roth, Karlsruhe, Germany)) was added, and the samples were heated at 70 °C for 30 min. After allowing the samples to cool, 100 µL of TMSI *N*-trimethylsilylimidazole (trimethylsilyation reagent, ≥98 % TCI, Zwijndrecht, Belgium) was added, and the samples were heated again at 70 °C for 30 min. After allowing the samples to cool again, 200 µL of 1,1,2-trichloro-1,2,2-trifluororethane (≥99.8 %, Promochem GmbH, Wesel, Germany) was added. The resulting clear solution was then analyzed by GC-MS using an HP 6890 gas chromatograph and a 5973 mass selective detector (Agilent/HP, Böblingen, Germany). A 30 m x 250 µm x 0.1 µm DB-5MS column (Agilent Technologies) was used. The temperature program was configured as follows: 140 °C (2 min), 3 °C/min up to 290 °C, 10 °C/min up to 340 °C (2 min). Then, 1 µL of the sample was injected at a split ratio of 20:1 (17.4 mL/ min split flow). The injector temperature was set to 360 °C. Helium was used as carrier gas at an average velocity of 35 cm/sec. The temperature of the transfer line was set to 350 °C. The mass selective detector was operated in scan mode with a solvent delay of 3 min. The mass range was *m*/*z* 50-800 and 2 scans/min. The temperature of the MS source was set to 230 °C, and that of the quadrupole was set to 150 °C.

Cytotoxicity analysis via resazurin assay

IHKE-cells were seeded in 96-well plates with 3750 cells/well and cultivated for 24 h. The cell culture medium was then replaced with serum-free medium (DMEM/F-12 medium supplemented with HEPES (15 mM), penicillin (100,000 U/L), and streptomycin (100 mg/L)), and the cells were incubated for 24 h. The compounds of interest (DCIT and CIT) were dissolved in ACN (10 mM stock solutions). A serial dilution in the range of 500 nM to 100 µM (500 nM, 1 µM, 2 µM, 5 µM, 10 µM, 20 µM, 50 µM, 100 µM) was freshly prepared in serum-free medium with a final concentration of 1% ACN in all dilutions. After removing the serum-free medium, IHKE-cells were incubated with 100 µL per well of the aforementioned serial dilutions with 1% ACN serving as negative control and 10 µM T-2 toxin as positive control.

After incubating the IHKE cells with the test compounds for 24 h, 10 µL 440 µM resazurin solution was added to each well, and cells were again incubated for 2 h at 37 °C, 5% CO_2 in the dark. Subsequently, the reduction of resazurin to resofurin was analyzed using the fluorescence at $\lambda_{exc} = 544$ nm and $\lambda_{emi} = 590$ nm by a microplate reader (Infinite M200Pro, Tecan Austria GmbH, Männedorf, Switzerland). Cellular cytotoxicity was determined in six technical replicates of three biological replicates each (n = 6 x3). After subtraction of cell-free blank values, the cell viability was calculated as the quotient of the detected fluorescence of the test substance and the negative control. Cytotoxicity data for each investigated concentration of CIT and DCIT was statistically evaluated using unpaired, heteroscedastic Student's t-test relative to the negative control.

Table S1. MS-source parameters for the Sciex QTRAP 6500.

**ESI source**	Ion Drive, Turbo V Source	
**Source parameters**	Polarity	positive
	Ion spray voltage	2500 V
	Curtain gas	45 psi
	Temperature	550 °C
	Ion source gas 1	60 psi
	Ion source gas 2	55 psi
	Cell entrance potential	10 V
	Cell exit potential	11 V

Table S2. Detailed MS-parameters for all scheduled multiple reaction monitoring (sMRM) transitions for CIT and different degradation products of CIT.

Analyte	Q1 mass [*m/z*]	Q3 mass [*m/z*]	Exp. RT[c] [min]	Analysis window [sec]	DP[d] [V]	CE[e] [V]	CXP[f] [V]
**CIT**	251.1	115[a]<br>233[b]<br>215[b]	5.05	60	56<br>56<br>75	65<br>23<br>32	11<br>11<br>13
**$^{13}C^{3}$-CIT**	254.1	118<br>236<br>218	5.05	60	56<br>56<br>75	65<br>23<br>32	11<br>11<br>13
**DCIT**	207.1	174.1[a]<br>189.1[b]<br>143.1[b]	2.80	60	66	34<br>21.5<br>30	16<br>16<br>11
***m/z* 395 A/ B**	395.2	291.2[a]<br>377.2[b]<br>306.2[b]	6.25	120	56	64<br>9.2<br>41.5	22<br>10<br>18
***m/z* 425**	425	189[a]<br>207[b]<br>174[b]	7.95	60	130	47	11
**DH-CIT**	267.1	249.1[a]<br>231.1[b]<br>203[b]	5.1	60	45<br>51<br>51	35<br>39<br>32	11<br>14<br>14
**Phenol A[g]**	197.1	123	6.2	780	45	35	11
**Phenol A acid[g]**	241.1	223.1<br>205.1	3.88	60	45	35	11
**CIT H1[g]**	427.2	219.1<br>91	5.4	150	54<br>32	31<br>88	11
**CIT H2[g]**	225.1	179.1	4.2	60	45	35	11
**Dihydro-citrinin[g]**	253.1	235<br>193.1	5.2	150	45	35	11
**Dicitrinin A[g]**	381.2	363.2<br>338.2	4.45	210	45	35	11
**Dicitrinin C[g]**	393.2	360.1<br>207.1	3.16	60	45	35	11
***m/z* 411[g]**	411.2	393.2<br>205	3.2	60	54<br>63	29<br>94	11
***m/z* 413[g]**	413.2	207.1<br>219	5.2	120	37<br>70	35<br>25	11
***m/z* 457[g]**	457.2	219.4<br>207.2	7.07	60	57<br>54	29	11
***m/z* 601[g]**	601.3	219.1<br>191	6.7	90	49<br>50	34<br>64	11
***m/z* 615[g]**	615.3	393.2<br>217.3<br>189.4	4.9	120	46<br>43<br>52	34<br>64<br>67	11

[a] Quantifier
[b] Qualifier
[c] Expected retention time, as they were used for sMRM
[d] Declustering potential
[e] Collision Energy
[f] Cell Exit Potential
[g] No standards or isolated compounds available

Table S3. Detailed MS-parameters for all multiple reaction monitoring (MRM) transitions for CIT and reaction products of CIT and starch.

Analyte	Q1 mass [*m/z*]	Q1 mass [*m/z*]	Dwell time [msec]	DP[c] [V]	CE[d] [V]
**CIT**	251.1	115[a]	20	56	65
		233[b]		56	23
***m/z* 369**[e]	369.2	219.1[a]	40	49	38
		303.1[b]		47	25
***m/z* 351**[f]	351.1	333	30	50	21
		303.1		89	22
		219.1		56	33
		315.1		55	25
		287.1		55	25
		259.1		55	25

[a] Quantifier
[b] Qualifier
[c] Declustering potential
[d] Collision Energy
[e] Since no standards or isolated compounds are available, the CIT-calibration and the quantifier of *m/z* 369 (reaction products **A**) are used for the semi-quantitative estimation of the amounts formed in the samples
[f] MRM transition was identified in model experiments, MRM transitions could not be identified in biscuit samples

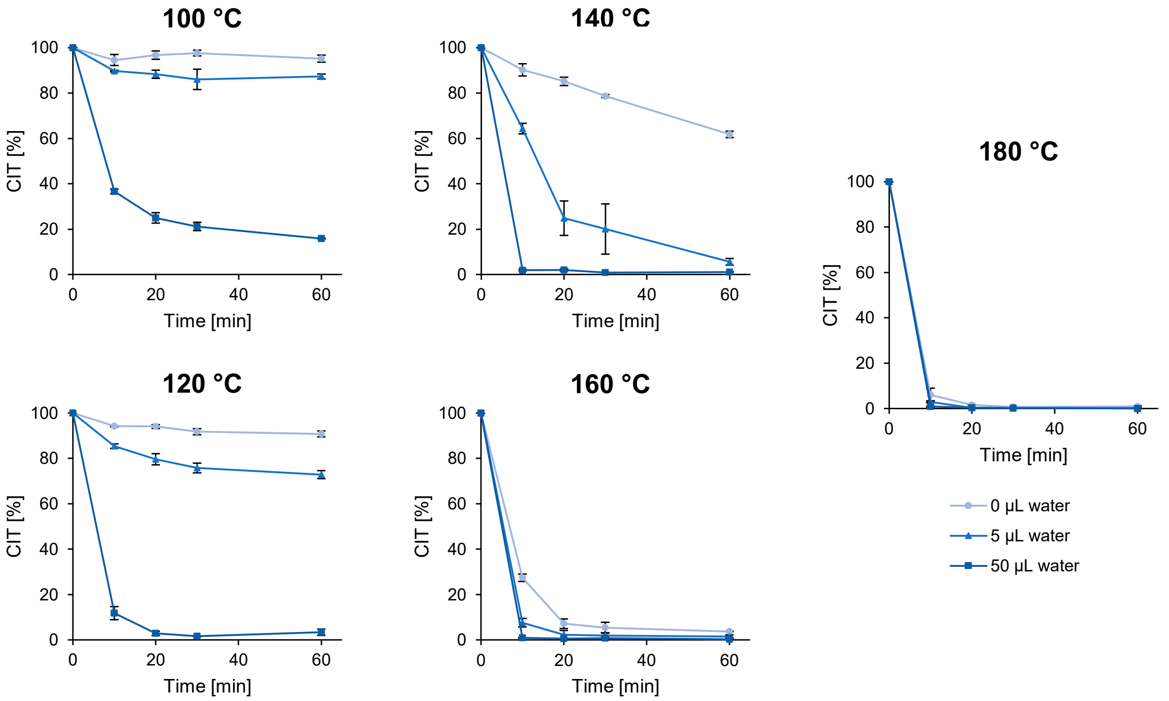

Figure S1. Thermal stability of CIT during heating for different times (10, 20, 30, 60 min) and temperatures (100, 120, 140, 160, 180 °C) with and without addition of different amounts of water (0 µL, 5 µL, 50 µL).

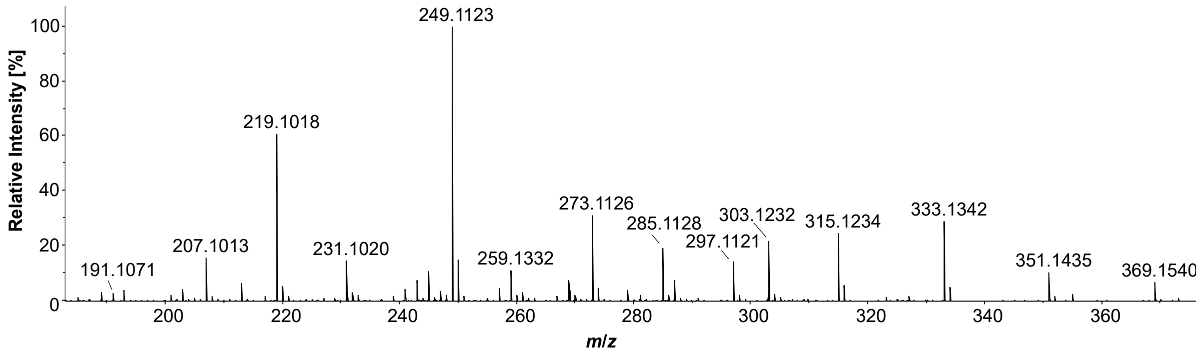

Figure S2. CIT heated together with α-D-glucose at 160 °C for 10 min. HRMS product ion spectrum of the reaction product **A** with *m*/*z* 369.1540, retention time 4.68 min, CE 31.7 eV ($[M+H]^+$, $C_{18}H_{24}O_8$).

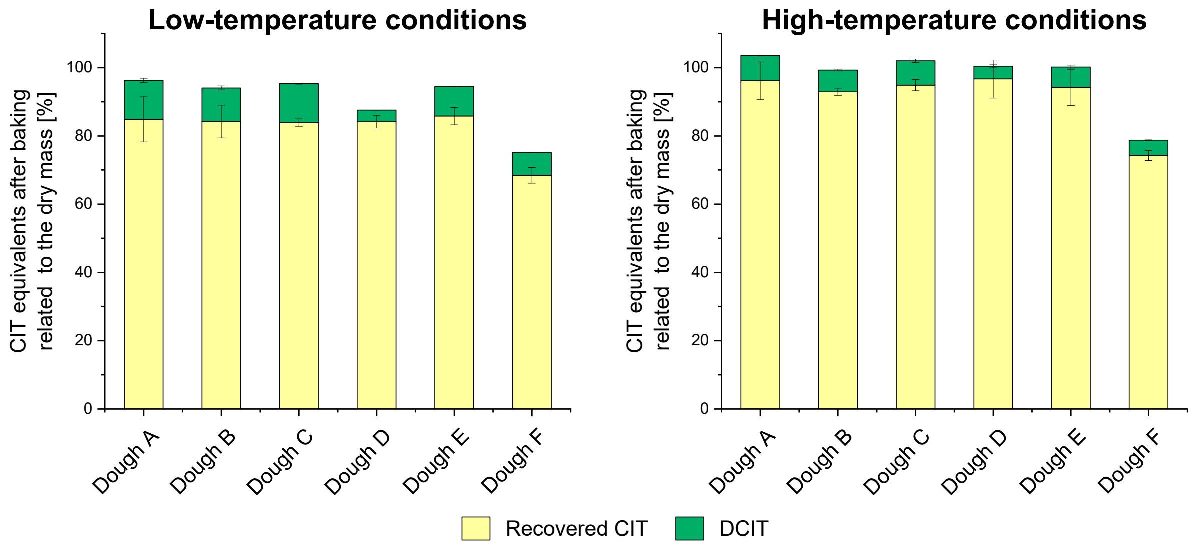

Figure S3. CIT degradation during biscuit-making in the different doughs (dough A-B: variation of the water content; dough C-D: variation of the sugar used; dough E-F: variation of the wheat flour type used). Low-temperature: 180 °C, 20 min; high-temperature: 220 °C, 10 min.

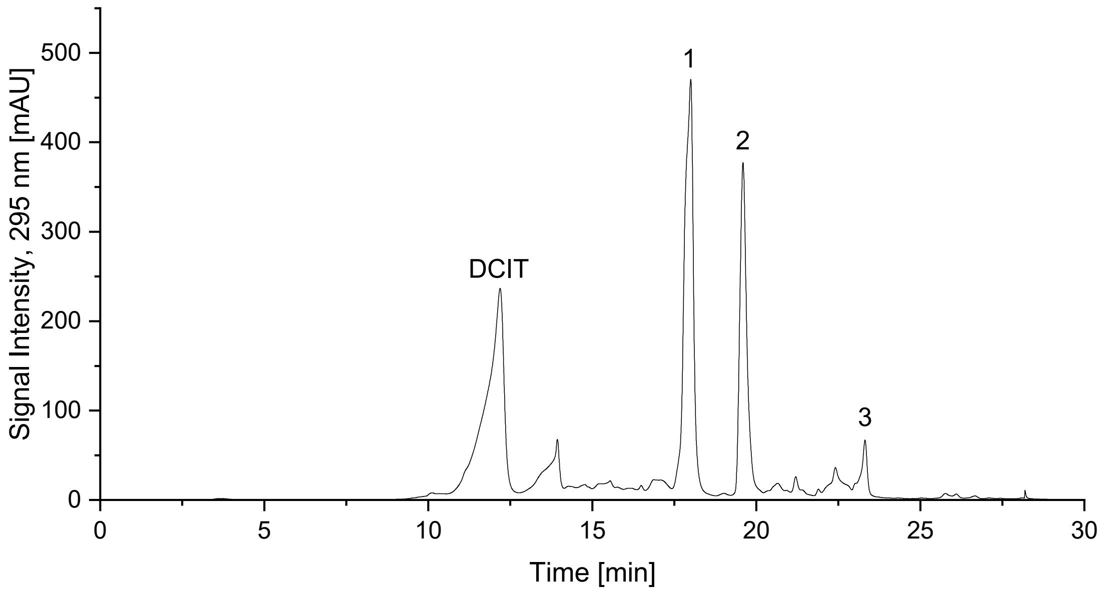

Figure S4. Chromatogram of the semi-preparative HPLC-UV for the purification of the degradation products of CIT. In addition to DCIT, peaks **1**, **2**, and **3** were also collected and further characterized by HPLC-DAD-ESI-QTOF: **1**: *m*/*z* 395.1864, **2**: *m*/*z* 395.1857, **3**: *m*/*z* 425.1956.
